# LncRNA PTPRG-AS1 Promotes the Metastasis of Hepatocellular Carcinoma by Enhancing YWHAG

**DOI:** 10.1155/2021/3624306

**Published:** 2021-11-28

**Authors:** Gaoyang Chen, Zhisheng Zhang, Yan Li, Lu Wang, Yanqing Liu

**Affiliations:** ^1^The First Clinical Medical College of Nanjing University of Traditional Chinese Medicine, Nanjing, Jiangsu 210023, China; ^2^Oncology Department, The Second People's Hospital of Taizhou City, Taizhou, Jiangsu 225511, China

## Abstract

**Objectives:**

Hepatocellular carcinoma (HCC) is one of the most common malignant tumors. LncRNA PTPRG-AS1 (PTPRG-AS1) has been confirmed to function as a regulator in various cancers, whose function during HCC tumorigenesis is still not clear now. Thus, we aim to dig out the biological function and its mechanisms of PTPRG-AS1 in HCC.

**Methods:**

PTPRG-AS1 relative expression in tissues and cells was detected and analyzed using real-time quantitative PCR (qRT-PCR). Subcellular distribution of PTPRG-AS1 was examined by FISH experiments. The effects of PTPRG-AS1 in the growth of HCC were studied by in vitro CCK-8 experiments, transwell invasion experiments, and in vivo xenograft tumor experiments. Dual-Luciferase reporter assay was performed to verify the interaction between PTPRG-AS1 and miR-199a-3p or miR-199a-3p and its target gene, YWHAG.

**Results:**

PTPRG-AS1 was upregulated in HCC tissues compared with adjacent normal tissues. We identified PTPRG-AS1 mainly localized in the cytoplasm of HCC cells. Downregulation of PTPRG-AS1 suppressed HCC progression, while overexpression of PTPRG-AS1 showed the opposite effects. Furthermore, PTPRG-AS1 served as a miR-199a-3p sponge and positively regulated YWHAG expression. Besides, PTPRG-AS1 could promote HCC through miR-199a-3p/YWHAG axis.

**Conclusions:**

Taken together, we demonstrated PTPRG-AS1 may serve as a ceRNA and reversely regulates the expression of miR-199a-3p, thus facilitating HCC tumorigenesis and metastasis, which is expected to provide new clues for the treatment of HCC.

## 1. Introduction

Hepatocellular carcinoma (HCC) accounts for more than seven percent of primary liver cancers and is widespread in various countries, causing serious social impact and a heavy economic burden [1, 2]. HCC-derived deaths have been indicated as the second reason for death caused by cancer in humans due to its high incidence illustrated by the World Health Organization (WHO) [3]. Every year, more than 500,000 people worldwide are diagnosed with HCC. The latest data show that there were approximately 782,500 new cases of HCC and 745,500 deaths in 2012, of which about half of the above numbers occurred in China [4]. HCC is considered an extremely complex process involving proliferation, migration, and/or apoptosis in the hepatocytes [5, 6]. At present, surgical resection or liver transplantation is the most effective cure for patients with liver cancer [7, 8]. Nonetheless, patients with advanced liver cancer cannot undergo these surgical interventions and liver transplantation [9, 10]. Currently, tremendous efforts have been made in exploiting molecular markers in HCC, and some achievements have been made [11–13]. The underlying mechanisms largely remained unclear. In other words, it is very urgent to find more effective markers and deepen their mechanisms underlying HCC carcinogenesis and progression.

Long noncoding RNAs (lncRNAs) are a subfamily of noncoding RNAs, usually longer than 200 nts in length, and have recently caused extensive attention by researchers worldwide because of their extensive role in various diseases [14, 15]. Recent research elaborated that lncRNAs can act as a “sponge” and compete for the binding of miRNAs of other genes, thereby reducing miRNA's regulatory effect on targeted mRNA, which is also known as the mechanism of “competing endogenous RNA” (ceRNA) [16–19]. In recent years, much evidence has elaborated that lnRNAs play an increasingly important role in progression of liver cancer by changing multiple cellular processed the including proliferation or invasion [20–23]. Various HCC-interrelated lncRNAs have been elaborated to own abnormal expression pattern and involvement in the cancer phenotype. For example, lncRNA-D16366 is reduced in HCC, which may be an independent therapeutic indicator of the disease [24]. LncRNA HEIH was highly expressed and suppress tumor growth and metastasis in HCC tissues and may be a prospective target for HCC therapy [25]. LncRNA PTPRG antisense RNA 1 (PTPRG-AS1), a rarely reported novel lncRNA, was identified as an abnormal expression for the first time in breast cancer by Mostafa et al. [26]. Subsequent experiment demonstrated that the PTPRG-AS1 expression were dramatically higher in epithelial ovarian cancer (EOC) tissues and may serve as a novel molecular marker for EOC patients [27]. Functional experiments suggest PTPRG-AS1 acts as a miRNA “sponge” to regulated protein regulator of cytokinesis 1 (PRC1) in nasopharyngeal carcinoma [28]. However, the effect model of PTPRG-AS1 in HCC is still mostly uncharacterized up to date. We elaborated that PTPRG-AS1 was highly expressed in HCC tissues for the first time and expected to regarded as a novel molecular marker for HCC patients.

In this study, we analyzed the expression of PTPRG-AS1 in human HCC tissues and paracancer tissues. Subsequently, functional experiments were performed to verify the carcinogenic effect of PTPRG-AS1. The regulatory relationship between PTPRG-AS1 and miR-199a-3p was further studied. This study may provide new biomarkers for the treatment of liver cancer. Our study may also provide new insights into clinical treatment and further intervention targets for HCC.

## 2. Materials and Methods

### 2.1. Patient Tissue Samples

We collected 30 cases of hepatocellular carcinoma (HCC) tumor tissues, along with matched adjacent normal tissues at our hospital. Inclusion criteria: (1) preoperative radiotherapy and chemotherapy not performed, (2) pathological examination confirming primary liver cancer, and (3) complete data. Exclusion criteria: (1) reject participants, (2) patients suffering from other malignant tumors, and (3) patients suffering from serious heart, liver, kidney, and other important organ diseases. The diagnosis basis of liver cancer refers to Chinese Society of Clinical Oncology (CSCO) guidelines for the diagnosis and treatment of primary hepatocellular carcinoma (HCC). All experiments were approved by the Research Scientific Ethics Committee of the second People's Hospital of Taizhou City. All experiments were conducted with informed consent signed by all diagnosed patients.

### 2.2. Cell Culture

SMMC-7721 (human hepatoma cell line), HepG2 (human hepatoma cell line), Huh-7 (human hepatoma cell line), PLC-PRF-5 (human hepatoma cell line), and THLE-3 (human liver epithelial cells) cells were purchased from American Type Culture Collection (ATCC, Manassas, VA, USA). All cells were cultured in normal-glucose DMEM medium (Gibco, Rockville, MD, USA) containing 10% fetal bovine serum (FBS, Gibco) and kept in an incubator (Thermo Fisher Scientific, Waltham, MA, USA) containing 5% CO_2_ under 37°C.

### 2.3. Fluorescence In Situ Hybridization (FISH) Assay

FISH assays were employed using the Fluorescence *In Situ* Hybridization kit (RiboBio, Guangzhou, Guangdong, China). Cells were seeded on slides and fixed in 4% paraformaldehyde (PFA) at room temperature for half an hour. Then, cells were treated with 0.5% Triton X-100 on ice for 15 min to enhance membrane permeability. Subsequently, cells were mixed with hybridization buffer containing FISH probes for half an hour under 60°C. After washing off the residual reagent, the slides were dehydrated, and DAPI (Promega, Madison, WI, USA) was employed for staining nucleus. The laser-scanning confocal microscope was employed to observe images (Leica Microsystems, Germany).

### 2.4. Cell Transfection

The short hairpin RNAs (shRNAs) for PTPRG-AS1 (sh-PTPRG-AS1) and their negative control (sh-NC) and the miR-199a-3p mimics, mimics-NC, miR-199a-3p inhibitor, and inhibitor-NC were designed by GenePharma Co., Ltd. (Shanghai, China). About 2 × 10^5^ cells were cultured in six-well plates with 2 mL complete medium, and transfection was routinely performed with the help of Lipofectamine 3000 (Invitrogen, Carlsbad, CA, USA) when they were 80% confluent. qRT-PCR was employed to detect the transfection efficiency after 48 hours of transfections.

### 2.5. RNA Isolation and RT‐qPCR Assay

RNA was extracted from corresponding tissues and cell lines using TRIzol reagent and then synthesized into cDNA by the corresponding reverse transcription kit (Invitrogen, Carlsbad, CA, USA). Subsequently, quantitative PCR was done using SYBR Green reagent (Invitrogen) on the 7500 Fast Real-Time System (Thermo Fisher Scientific, Waltham, MA, USA) to detect the RNA expression levels. The results were standardized to GAPDH and U6. The relative quantification of indicated genes were normalized through the 2^−ΔΔCt^ method. All genes were assayed at least in triplicate. The primer information is lists in Table 1.

### 2.6. Cell Viability Assay

Cell viabilities were estimated by the CCK‐8 detection kit (Apexbio, Houston, USA). Briefly, treated HepG2 and PLC-PRF-5 cells were seeded into 96-well plates (4 × 10^3^ cells/well) and incubated for about 12 h. 10 *μ*L CCK‐8 solutions was instilled to each well 24, 48, 72, and 96 hours later respectively, and cells were incubated for another 2 h under 37°C. The absorbance value was detected under 450 nm wavelength with a microplate reader (MultiskanEX, Lab systems, Helsinki, Finland).

### 2.7. EdU Staining

We seeded the transfected PLC-PRF-5 cell and HepG2 cell into 96-well plates until cell attachment and detected DNA synthesis using EdU detection Kit (RiboBio, Guangzhou, China). Briefly, we added reagent A to each well (100 *μ*L/well, 1 diluted to 1000 with media) and made them react at 37°C for 120 min. Then, we added PBS solution containing 4% PFA to each well and incubated at room temperature for 30 minutes for cell fixation. After that, we added 50 microliters of glycine solution (2 mg/mL) to each well to neutralize the residual paraformaldehyde. We discarded the solution and added PBS to wash. 100 microliters of penetrating agent (PBS solution with 0.5% Trixon-100) was supplemented to each well to enhance the cell membrane permeability. 1X Apollo staining reaction solution was supplemented to each well for 30 min in the dark at room temperature. We used PBS containing 0.5% Trixon-100 to rinse cells and then washed them with methanol to reduce the staining background. Finally, we employed the Hoechst solution for nuclear coloration and washed it three times with PBS. The staining was observed under a microscope (Nikon, Tokyo, Japan) and photographed immediately after all procedure completion.

### 2.8. Transwell Invasion and Migration Assay

In terms of transwell invasion assay, approximately 5 × 10^3^ treated PLC-PRF-5 or HepG2 cells were resuspended and placed in the upper space of a transwell system (8 *μ*m pore size, Corning, Cambridge, USA) with a Matrigel-coated membrane (BD Bioscience, San Jose, USA), containing medium without serum. Lower chambers were supplemented with a 100% complete culture medium. Consequently, hungry cells will penetrate from the upper to the bottom, attaching themselves to the membrane below. Afterward, the upper layer was removed, while the cells in the lower layer were retained for subsequent analysis, employing 4% PFA to fix the retained cells and using 0.1% crystal violet solution to stain for 30 min to evaluate invaded cell numbers. For the transwell migration experiment, the procedure was described as invaded assay above only without Matrigel-coated membrane. We randomly selected five mirror views to perform cell counts under a 200× microscope (Leica Microsystems, Germany).

### 2.9. HCC Tumor Xenograft In Vivo

12 athymic male nude mice of BALB/c aged about 6 weeks old were purchased from the National Experimental Animal Center (Beijing, China) and kept in a sterile environment with stable humidity and temperature. All animal procedures have been approved by the Animal Research Ethics Committee of the Second People's Hospital of Taizhou City. Approximately, 1 × 10^7^ PLC-PRF-5 after transfection with LV-NC (*n* = 6) and LV-shPTPRG-AS1 (*n* = 6) were diluted in 100 *μ*L medium, mixed well with a pipette, and then hypodermically injected into the dorsal skin of nude mice. We used a digital caliper to measure tumor volume on the 7^th^, 14^th^, 21^st^, and 28^th^ day after injection and calculated using the following formula: tumor volume = 4*π*/3 × (width/2)^2^ × (length/2). When all procedures were completed, nude mice were euthanatized and tumor tissues were isolated for tumor weight examination.

### 2.10. Dual-Luciferase Reporter Assay

Wild-type PTPRG-AS1 (wt-PTPRG-AS1) or YWHAG (wt-YWHAG) and mutant PTPRG-AS1 (mut-PTPRG-AS1) or YWHAG (mut-YWHAG) were employed to insert into pGL3 vector (Promega, Madison, WI, USA). Subsequently, luciferase constructs and miR-199-3p mimics or negative control were cotransfected into cells using Lipofectamine 3000 (Invitrogen). Luciferase activities were evaluated through the dual-luciferase assay system (Promega) 48 h after transfection.

### 2.11. Histology

Tumor tissue slides were stained using hematoxylin and eosin (H&E) to observe the changes in metastases numbers due to PTPRG-AS1 knockdown. 5 *μ*m microsections were prepared and fixed by infusing 4% PFA and then subsequently stained with H&E to assess tumor metastases numbers. For immunohistochemical (IHC) assay, slides were also fixed with 4% PFA, using 0.1% Triton X-100 in PBS solution to penetrate. The slides were blocked using PBS solution supplemented with 5% bovine serum albumin to remove specific background staining. After cleaning and removing residual reagent, Ki67 primary antibody (ab15580, 1 : 200, Abcam, Cambridge, MA, USA) was employed to cover sections overnight at 4°C. Primary antibody was washed away with PBS, and the samples were incubated with a specific secondary antibody for 1 h under room temperature condition and imaged using a light microscope.

### 2.12. Statistical Analysis

Statistical analysis was performed employing GraphPad Prism 7.0 software. Mean ± standard deviation (SD) was used to describe all quantitative experimental data. Two-tailed student's *t*-test was employed to evaluate the difference between the two groups. One-way ANOVA analysis followed by Tukey's multiple comparison test was used to compare the difference between multiple groups. *P* < 0.05 was considered statistically significant.

## 3. Results

### 3.1. PTPRG-AS1 Was Intensified and Correlated with Poor Prognosis in HCC

**Figure 1:**
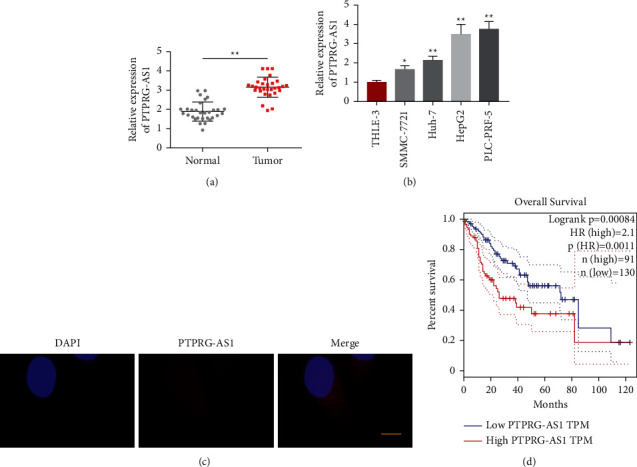
PTPRG-AS1 was upregulated in hepatocellular carcinoma tissues. qRT-PCR was used to test the expression level of PTPRG-AS1 in liver cancer tissues (*n* = 30) (a) and different hepatoma cell lines (b). (c) FISH analysis of PTPRG-AS1 (nuclei were stained with blue, and PTPRG-AS1 was stained with red; scale bar: 5 *μ*m). (d) Kaplan–Meier curves display the estimated survival probability in HCC patients with high expression and low expression of PTPRG-AS1. All experimental data are carried out 3 times, and mean ± standard deviation was employed to represent experimental data. ^*∗*^*P* < 0.05, ^∗∗^*P* < 0.01.

To elaborate the specific function of PTPRG-AS1 in HCC, qRT-PCR analysis was employed to detect the quantification of PTPRG-AS1 in human HCC tissues obtained from 30 HCC patients and their adjacent normal tissue. The result exhibited that PTPRG-AS1 quantification was intensified if we contrasted this to neighboring tissues (Figure 1(a)). Then, the PTPRG-AS1 quantification in 4 different HCC cell lines was estimated. We found that PTPRG-AS1 quantification was intensified in HepG2 and PLC-PRF-5 compared with that in SMMC-7721 and Huh-7 (Figure 1(b)). In addition, FISH assays were carried out to demonstrate the localization of PTPRG-AS1 in the nuclei and cytoplasm.  Figure 1(c) shows that PTPRG-AS1 was predominately located in the cytoplasm. Next, we divided patients into high or low expression groups according to PTPRG-AS1 quantification. We used the Kaplan–Meier survival analysis to make the overall survival (OS) curves to explore the consequence of PTPRG-AS1 in clinical prognosis. The results showed that patients with high PTPRG-AS1 expression quantification had shorter OS than patients with low PTPRG-AS1 (Figure 1(d)). These results demonstrated that a high PTPRG-AS1 expression level was significantly associated with poor HCC outcomes.

### 3.2. PTPRG-As1 Influences HCC Tumor Proliferation and Invasion In Vitro

**Figure 2:**
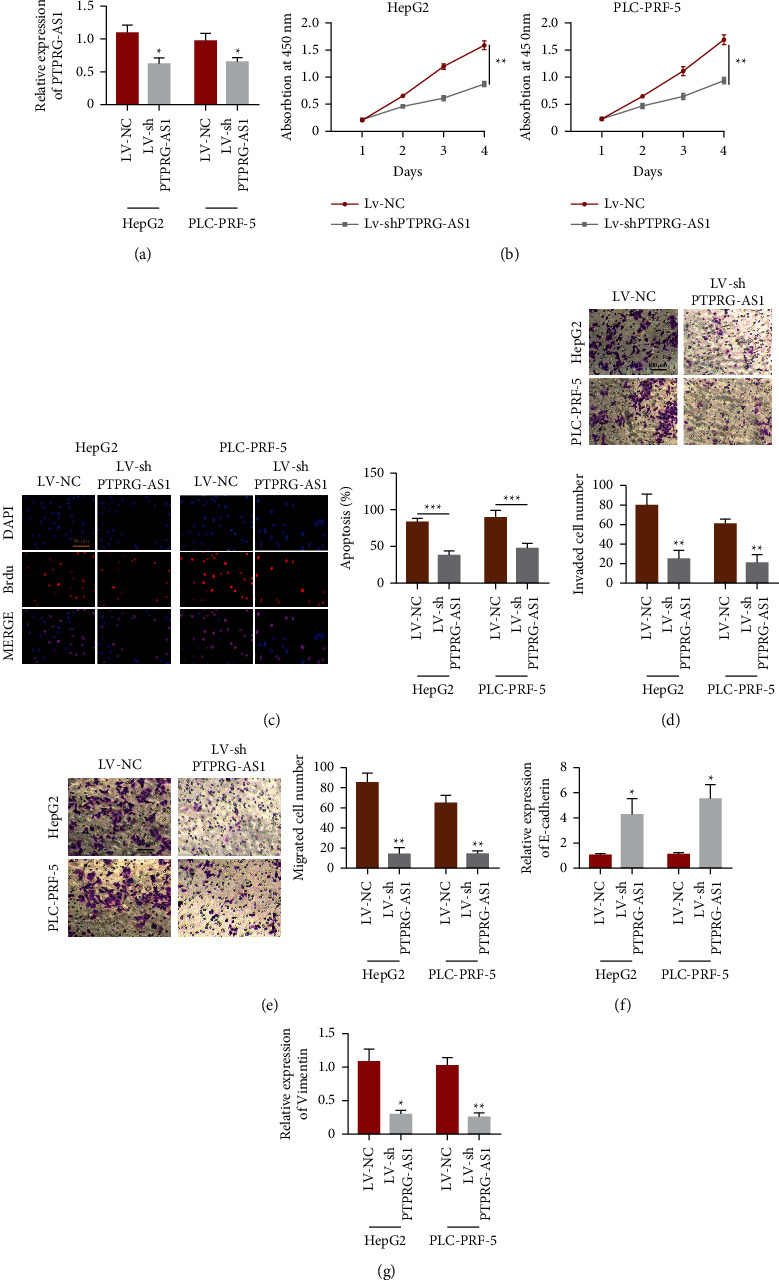
Downregulation of PTPRG-AS1 attenuated proliferation and invasion of hepatocellular carcinoma in vitro. (a) qRT-PCR was employed to examine the quantification of PTPRG-AS1 in HepG2, PLC-PRF-5 transfected with LV-shPTPRG-AS1 and LV-NC. (b) CCK-8 assay was used to investigate cell viabilities of HepG2 and PLC-PRF-5 transfected with LV-shPTPRF-AS1 and LV-NC. (c) EdU analysis was employed to test the proliferation of HepG2 and PLC-PRF-5 transfected with LV-shPTPRG-AS1 and LV-NC. (d) Transwell assay was used to estimate the invaded cell numbers of HepG2 and PLC-PRF-5 transfected with LV-shPTPRG-AS1 and LV-NC. (e) Transwell assay was used to estimate the migrated cell numbers of HepG2 and PLC-PRF-5 transfected with LV-shPTPRG-AS1 and LV-NC. mRNA quantification of E-cadherin (f) and vimentin (g) in HepG2 transfected with LV-shPTPRG-AS1 and LV-NC were examined by qRT-PCR analysis. ^*∗*^*P* < 0.05, ^∗∗^*P* < 0.01, ^∗∗∗^*P* < 0.001.

Next, we knocked down PTPRG-AS1 using siRNA transfection technology (Figure 2(a)). Subsequent cell proliferative experiment revealed that downregulation of PTPRG-AS1 restrained the growth of HepG2 and PLC-PRF-5 (Figure 2(b)). Furthermore, the EdU assay showed that the proliferation potential of HepG2 and PLC-PRF-5 was all impaired upon downregulation of PTPRG-AS1 (Figure 2(c)). To further investigate the biological effects of PTPRG-AS1 in tumor growth in vitro, transwell invasion and migration were performed. Moreover, we found that inhibition of PTPRG-AS1 led to a prominent decrease in invaded cell numbers (Figure 2(d)) and migrated cell numbers (Figure 2(e)). In addition, we employed qRT-PCR to examine the quantification of E-cadherin and vimentin in HCC cells. E-cadherin was intensified upon downregulation of PTPRG-AS1 (Figure 2(f)). However, vimentin was attenuated (Figure 2(g)). These findings showed that downregulation of PTPRG-AS1 could inhibit proliferation and migration in HCC.

**Figure 3:**
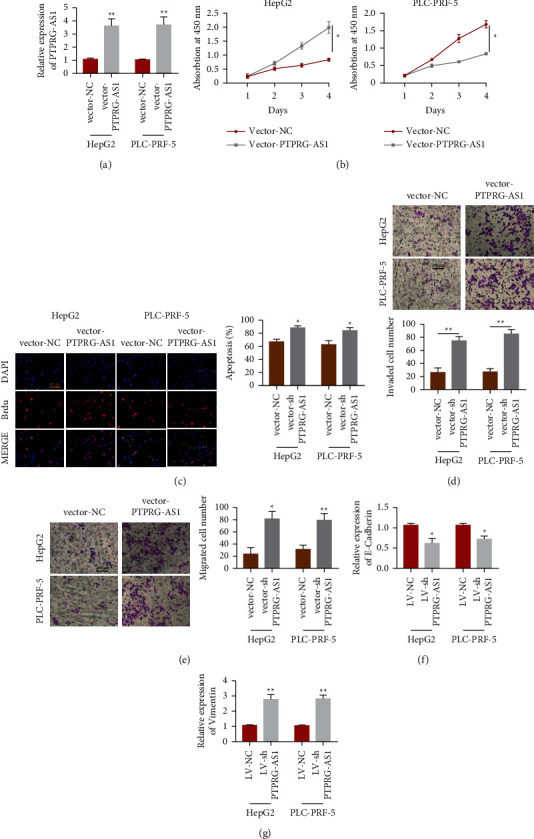
Overexpression of PTPRG-AS1 promoted HCC proliferation and invasion in vitro. (a) qRT-PCR was employed to estimate the quantification of PTPRG-AS1 in HepG2 and PLC-PRF-5 transfected with vector-PTPRG-AS1 and vector-NC. (b) CCK-8 assay was employed to investigate the cell viabilities of HepG2 and PLC-PRF-5 cells transfected with vector-PTPRF-AS1 and vector-NC. (c) EdU analysis was employed to detect the proliferation of HepG2 and PLC-PRF-5 cells transfected with vector-PTPRG-AS1 and vector-NC. (d) Transwell assay was used to estimate invaded cell numbers of HepG2 and PLC-PRF-5 transfected with vector-PTPRG-AS1 and vector-NC. (e) Transwell assay was used to estimate migrated cell numbers of HepG2 and PLC-PRF-5 transfected with vector-PTPRG-AS1 and vector-NC. mRNA quantification of E-cadherin (f) and vimentin (g) in HepG2 cell transfected with vector-PTPRG-AS1 and vector-NC were examined by qRT-PCR analysis. ^*∗*^*P* < 0.05, ^∗∗^*P* < 0.01.

In order to further explore the function of PTPRG-AS1, we conducted overexpression analysis. As shown in Figure 3(a), we observed the successful overexpression efficiency of PTPRG-AS1 in HepG2 and PLC-PRF-5. Moreover, overexpression of PTPRG-AS1 dramatically promoted cell proliferation of HepG2 and PLC-PRF-5 (Figure 3(b)). Then, EdU assay showed that the proliferation potential of HepG2 and PLC-PRF-5 was all improved upon upregulation of PTPRG-AS1 (Figure 3(c)). Similarly, transwell invasion and migration were employed to detect cell growth in vitro. Furthermore, we found that upregulation of PTPRG-AS1 led to a prominent increase in invaded cell numbers (Figure 3(d)) and migrated cell numbers (Figure 3(e)). Moreover, PTPRG-AS1 expression increased the mRNA expression of vimentin (Figure 3(g)), whereas it decreased E-cadherin (Figure 3(f)). The above findings elaborated that the increased expression of PTPRG-AS1 could intensify proliferation and migration in HCC.

### 3.3. PTPRG-AS1 Acts as miR-199a-3p Sponge

**Figure 4:**
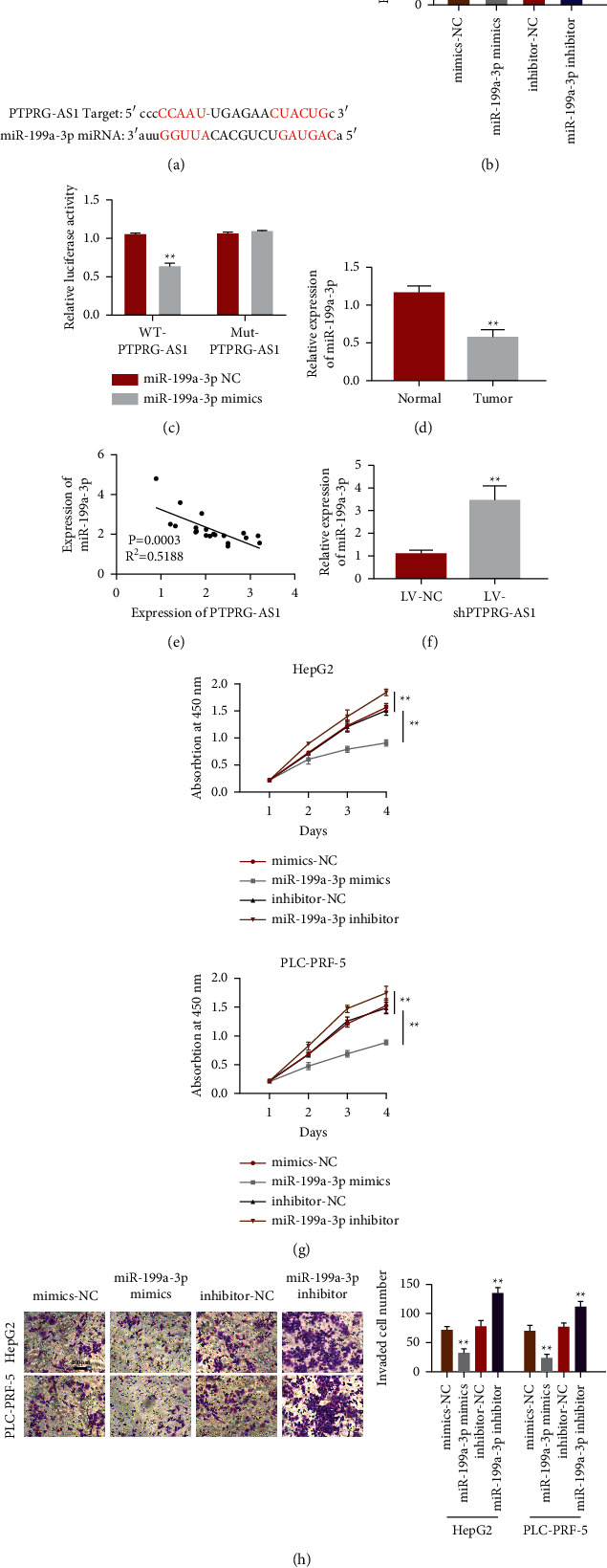
PTPRG-AS1 directly targeted miR-199a-3p. (a) starBase prediction of PTPRG-AS1 and miR-199a-3p sites. (b) qRT-PCR was employed to estimate the quantification of miR-199a-3p after transfection with mimics-NC, miR-199a-3p mimics, inhibitor-NC, or miR-199a-3p inhibitor. (c) Luciferase reporter analysis of the targeted binding between miR-199a-3p and PTPRG-AS1. (d) qRT‐PCR was employed to exam miR-199a-3p miRNA relative expression in HCC tumor tissue. (e) The linear correlations of PTPRG-AS1 and miR-199a-3p expression were demonstrated by Pearson analysis. (f) The relative level of miR-199a-3p in cell transfected with LV-shPTPRG-AS1 and LV-NC. (g) Overexpression of miR-199a-3p inhibited proliferation of HepG2 and PLC-PRF-5 indicated by CCK-8 assay. (h) Overexpression of miR-199a-3p attenuated invasion of HepG2 and PLC-PRF-5 detected by transwell assay. WT: wild-type. MUT: mutated. ^*∗*^*P* < 0.05, ^∗∗^*P* < 0.01.

To disclose the interaction between miR-199a-3p and PTPRG-AS1, starBase 2.0 database (http://starbase.sysu.edu.cn/) [29] was employed to predict their interaction. We found that miR-199a-3p was a promising miRNA target of PTPRG-AS1 (Figure 4(a)). Besides, miR-199a-3p relative expression after transfection with miR-199a-3p mimics increased notably (Figure 4(b)) and decreased notably after transfection of miR-199a-3p inhibitor (Figure 4(b)). Next, we used double luciferase reporter gene detection to explore whether miR-199a-3p could directly target PTPRG-AS1. The results showed that compared with the control group, luciferase activity was significantly decreased when miR-199a-3p was combined with PTPRG-AS1-WT. However, after mutation of ptPRG-AS1 binding site and addition of miR-199a-3p, luciferase activity did not change (Figure 4(c)). Inversely, miR-199a-3p relative quantification in tumor was decrease significantly if we contrasted this to neighboring tissues (Figure 4(d)). Moreover, Pearson's correlation analysis suggested PTPRG-AS1 quantification was significantly negatively correlated with miR-199a-3p expression in HCC (Figure 4(e)). Moreover, miR-199a-3p expression was enhanced with PTPRG-AS1 downregulation (Figure 4(f)). Subsequent CCK-8 assay revealed that upregulation of miR-199a-3p markedly suppressed proliferation of HepG2 and PLC-PRF-5 (Figure 4(g)). Similarly, transwell assay showed that upregulation of miR-199a-3p led to a significant reduction in invaded cell numbers, both HepG2 and PLC-PRF-5 (Figure 4(h)). Taken together, PTPRG-AS1 directly targeted miR-199a-3p.

### 3.4. PTPRG-AS1 Mediates miR-199a-3p/YWHAG Axis to Promote HCC

**Figure 5:**
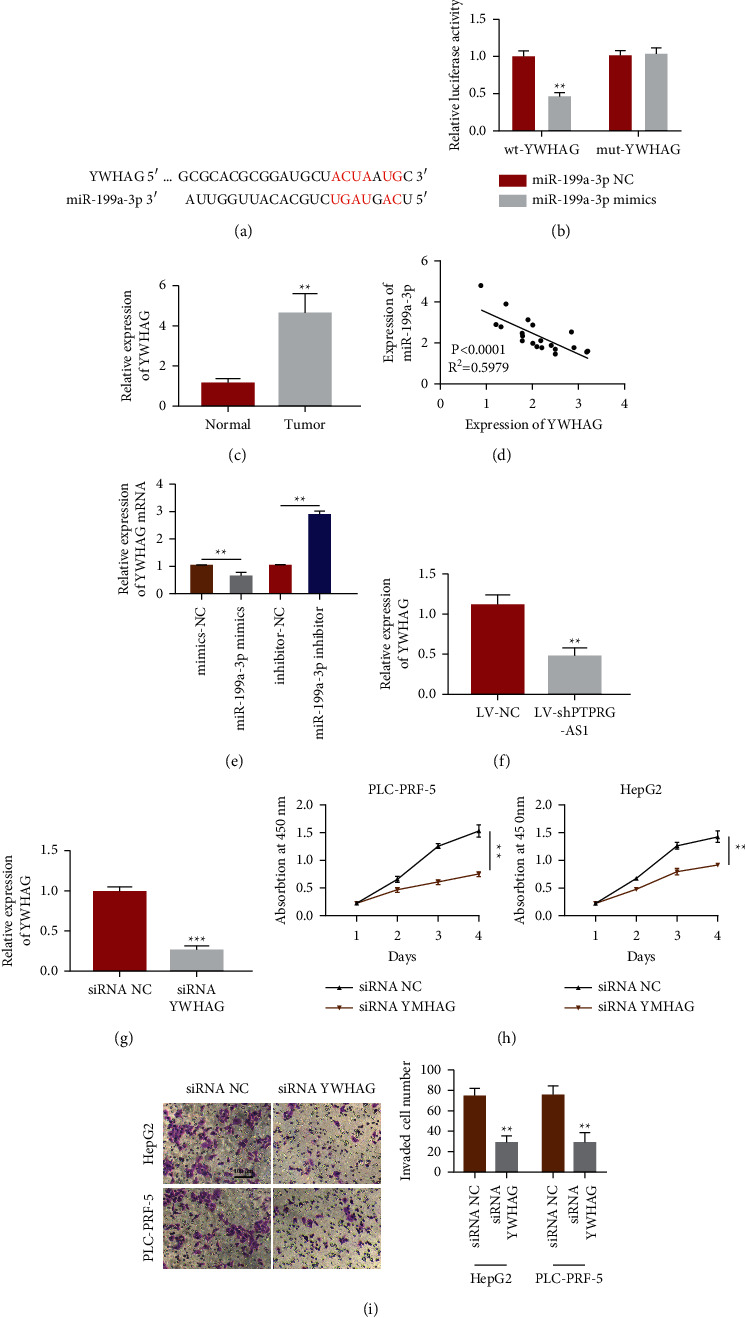
PTPRG-AS1 enhanced cell proliferation and invasion via miR-199a-3p/YWHAG axis. (a) YWHAG was the putative target of miR-199a-3p predicted by TargetScan. (b) Luciferase reporter analysis of the targeted binding between YWHAG and miR-199a-3p. (c) qRT‐PCR was employed to depict YWHAG mRNA relative quantification in HCC tumor tissue. The linear correlations of YWHAG and miR-199a-3p expression (d) were demonstrated by Pearson's analysis. (e) qRT‐PCR was employed to estimate the mRNA quantification of YWHAG after transfection with corresponding mimics or inhibitors. (f) qRT‐PCR was employed to estimate the mRNA quantification of YWHAG in cells transfected using LV-NC or LV-shPTPRG-AS1. (g) qRT‐PCR was used to detect YWHAG mRNA relative expression after transfection with siRNA-NC and siRNA YWHAG in cells. (h) Downregulation of YWHAG inhibited cell proliferation of HepG2 and PLC-PRF-5 cells detected by CCK-8 assay. (i) Downregulation of YWHAG inhibited cell invasion of HepG2 and PLC-PRF-5 cells detected by transwell assay. WT: wild-type. MUT: mutated. ^∗∗^*P* < 0.01, ^∗∗∗^*P* < 0.001.

Through the TargetScan database, we found YWHAG is a presumptive potential target of miR-199a-3p.  Figure 5(a) shows their matching binding sites. The luciferase reporter assay shows wild-type (wt) YWHAG could change the relative luciferase activity. In contrast, no obvious change in the luciferase activity was detected in the mut 3′-UTR of YWHAG (Figure 5(b)), indicating a direct interaction between YWHAG and miR-199a-3p. Furthermore, YWHAG relative quantification in the tumor was significantly heightened if we contrasted this to neighboring tissues (Figure 5(c)), which was opposite to that of miR-199a-3p. Furthermore, Pearson's correlation analysis suggested YWHAG was significantly negatively correlated with miR-199a-3p expression in HCC tissues (Figure 5(d)). qRT-PCR results uncovered that YWHAG relative expression decreased notably after transfection of miR-199a-3p mimics. However, YWHAG quantification was intensified after transfection with miR-199a-3p inhibitor (Figure 5(e)). Moreover, YWHAG expression was attenuated after transfection with sh-PTPRG-AS1 (Figure 5(f)). Next, we explore the effects caused by knocking down the expression of YWHAG to evaluate its biological function in HCC.  Figure 5(h) demonstrates that the inhibition was successful. Subsequent CCK-8 assay revealed that downregulation of YWHAG significantly restrained the growth of HepG2 and PLC-PRF-5 (Figure 5(h)). Furthermore, the transwell assay showed that the invasion potential of HepG2 and PLC-PRF-5 was all impaired upon downregulation of YWHAG (Figure 5(i)). Thus, PTPRG-AS1 mediated miR-199a-3p/YWHAG axis to promote HCC.

### 3.5. Downregulation of PTPRG-As1 Restrains HCC Cell Growth and Migration In Vivo

**Figure 6:**
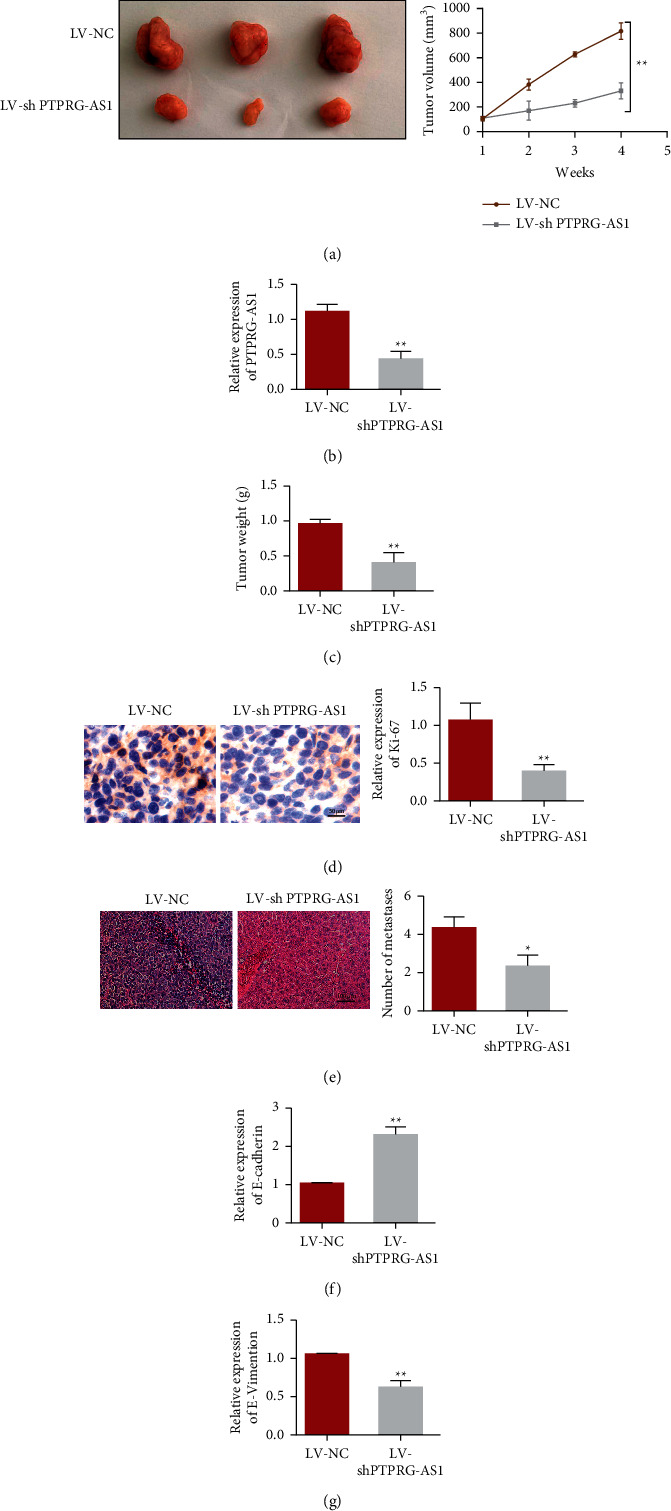
Downregulation of PTPRG-AS1 inhibited HCC proliferation and invasion in vivo. (a) Tumor image and volume in nude mice injected subcutaneously with PLC-PRF-5 cell transfected with LV-NC and LV-shPTPRG-AS1 (*n* = 6). (b) qRT‐PCR was employed to estimate miRNA quantification of PTPRG-AS1 in nude mice of the above groups. (c) Tumor weight in nude mice injected subcutaneously with PLC-PRF-5 cell transfected with LV-NC and LV-shPTPRG-AS1. (d) Ki67 staining of tissues in different groups. (e) Number of metastases after PTPRG-AS1 downregulation by HE staining. qRT-PCR was employed to depict mRNA expression of E-cadherin (f) or vimentin (g) in nude mice of above groups. ^*∗*^*P* < 0.05, ^∗∗^*P* < 0.01.

Furthermore, to determine the effects of PTPRG-AS1 on HCC in vivo, PTPRG-AS1 knockdown (LV-shPTPRG-AS1) or control (LV-NC) PLC-PRF-5 cells were injected subcutaneously into nude mice (*n* = 6).  Figure 6(b) demonstrates that the expression of PTPRG-AS1 was inhibited. The results uncovered that tumor volume and weight in mice injected with LV-shPTPRG-AS1 PLC-PRF-5 cells were notably reduced compared with the above index in control mice (Figures 6(a) and 6(c)). Ki67 staining is frequently used in oncology to estimate a tumor's proliferation index. Here, IHC results showed that downregulation of PTPRG-AS1 significantly inhibited Ki67 expression in nude mice, indicating a decreased proliferation level after PTPRG-AS1 downregulation (Figure 6(d)). Moreover, PTPRG-AS1 downregulation led to a decrease in the number of metastases in vivo (Figure 6(e)). qRT‐PCR delineated that E-cadherin quantification was upregulated in the tumor of LV-shPTPRG-AS1 group compared with the control group. However, vimentin was downregulated (Figures 6(f) and 6(g)).

## 4. Discussion

Hepatocellular carcinoma (HCC) is one of the six most prevalent cancer in the world [30], with the incidence rate of men much higher than that of women [31]. The number of deaths caused by liver cancer ranked second in the world among all deaths derived from cancer, accounting for 11% of deaths caused by cancer, only after lung cancer [32]. The five-year survival rate for liver cancer is only 14% in the United States, which is even lower in some underdeveloped areas [33]. Thus, finding effective tumor markers can reduce HCC recurrence rate and improve survival rate, further providing important guidance for clinical treatment [34–38].

Although recent studies have identified many novel HCC biomarkers, including lncRNAs [39–41], LncRNAs have been found to have functions in HCC development. Studies have indicated that the lncRNAs, MIAT [42], GABPB1 [43], and PDPK2P [44] influence tumor progression in HCC. Additionally, research revealed a relationship between PTPRG-AS1 and epithelial ovarian cancer [27] or osteosarcoma [45]. However, whether PTPRG-AS1 participates in the tumor progression of HCC has not been investigated. In the current research, we assessed the quantification of PTPRG-AS1 in HCC to find out whether they could be served as a novel prognostic biomarker for early detection and prediction of HCC. Our study showed that PTPRG-AS1 was a major participant in regulating the HCC tumorigenesis. Then, we investigated their underlying molecular mechanisms of how PTPRG-AS1 modulated the cell activities of HCC and found that upregulation of PTPRG-AS1 could strengthen HCC development, including replication proliferation and metastasis through downregulating miR-199a-3p. As a ubiquitous miRNA, miR-199a-3p is one of the miRNAs conspicuously overexpressed in liver tissues under normal conditions. It is downregulated in almost all HCC, and this downregulation is associated with poor prognosis [46, 47]. The antineoplasmic activity of miR-199a-3p in HCC has already been verified in past research using the mice model [48, 49]. Studies in recent years have shown that the synergistic regulation of multiple genes by specific miRNA is sophisticated in controlling the onset of diseases [50]. For example, miRNA-199a-3p weakened tumor progression in HCC by targeting *VEGFA*, *HGF*, or *MMP2* [51]. Moreover, miRNA-199a-3p could effectively decrease tumorigenesis in HCC, which may be related to ZHX1-dependent PUMA signals [52]. In this research, we have recognized that miR-199a-3p expression was attenuated in HCC and negatively associated with PTPRG-AS1 expression, which may be regarded as a potential target of PTPRG-AS1.

Micro-RNAs (miRNAs), a set of noncoding RNAs, exert the biological functions through directly binding mRNAs and consequently suppress their expression [53–56]. *YWHAG* gene resides on 7q11.23 and encodes 14-3-3*γ* protein, a member of the 14-3-3 family, which functions as a scaffolding protein to maintain the stability of multiprotein complex and therefore participates in a multitude of cellular processes as a regulatory molecule, including cell viability and apoptosis [57–60]. It is reported that stably overexpressed YWHAB aggravated the metastases of tumors and is expected to be adopted as a new signature molecule for HCC prognosis and provide potentially powerful clues for its treatment [61]. As a member of the 14-3-3 family, YWHAG was intensified in HCC if we contrasted this to neighboring tissues. Cell developments of both HepG2 and PLC-PRF-5 were all restrained by YWHAG knockdown. Moreover, it is predicted that matching sites exist between YWHAG and miR-199a-3p. YWHAG is a direct molecular binding to miR-199a-3p confirmed by corresponding luciferase activities. There are also shortcomings in this study. Whether there are other target genes of PTPRG-AS1 needs further study. In addition, more experimental studies are needed to translate the findings into clinical practice.

## 5. Conclusion

In summary, PTPRG-AS1 expression was upregulated in HCC in this study. The expression of miR-199a-3p was decreased. PTPRG-AS1 can be regarded as a miR-199a-3p “sponge” and significantly promote the occurrence of liver cancer by activating YWHAG expression. The results of this study suggest that PTPRG-AS1 can be used as a new molecular marker for the progression and diagnosis of liver cancer and provide new ideas for the treatment of liver cancer.

## Figures and Tables

**Table 1:** Primer for qRT-PCR analysis.

Gene	Forward sequence (5′-3′)	Reverse sequence (5′-3′)
GAPDH	TCAAGGCTGAGAACGGGAAG	TGGACTCCACGACGTACTCA
U6	CTCGCTTCGGCAGCACATATACT	CGCTTCACGAATTTGCGTGT
PTPRG-AS1	AAGCCAAGCAGTCAGAAGC	CAATGACCCCTTCATTGAC
miR-199a-3p	GCACAGTAGTCTGCACATTGG	GTGCAGGGTCCGAGGTATTC
E-Cadherin	GGATTGTCGGATTGGGAGAA	CATTCTGCTGCTTGAGGGTT
Vimentin	GATGTTTCCAAGCCTGACCT	CACTTCACAGGTGAGGGACT

## Data Availability

The data used to support the findings of this study are available from the corresponding author upon request.
